# A complex presentation and successful management of fistulizing perianal Crohn’s disease extending to the mid-back

**DOI:** 10.1093/jscr/rjad364

**Published:** 2023-06-21

**Authors:** Christopher Vazquez, Karishma Kodia, Bhuwan Giri, Nivedh Paluvoi

**Affiliations:** Division of Colon and Rectal Surgery, Department of Surgery, University of Miami Leonard Miller School of Medicine, Miami, FL, USA; Division of Colon and Rectal Surgery, Department of Surgery, University of Miami Leonard Miller School of Medicine, Miami, FL, USA; Division of Colon and Rectal Surgery, Department of Surgery, University of Miami Leonard Miller School of Medicine, Miami, FL, USA; Division of Colon and Rectal Surgery, Department of Surgery, University of Miami Leonard Miller School of Medicine, Miami, FL, USA

## Abstract

Fistulizing perianal disease is a debilitating complication present in nearly half of all patients diagnosed with Crohn’s disease. The majority of anal fistulas arising in these patients are complex. Treatment can be challenging with therapy often requiring both medical and surgical interventions with differing levels of symptomatic relief. Fecal diversion is an option after medical and surgical modalities have been exhausted but demonstrates limited efficacy. Complex perianal fistulizing Crohn’s disease is inherently morbid and can be difficult to manage. We present a case of a young male with Crohn’s, severe malnutrition and multiple perianal abscess with extensive fistula tracts up to his back; a planned fecal diversion was instituted to control sepsis and allow for wound healing and optimize medical therapy.

## INTRODUCTION

Crohn’s disease (CD) is a chronic inflammatory bowel disease that can occur throughout the entire gastrointestinal tract. Anal fistulae are a complication of CD secondary to anal crypt gland infection leading to abscess formation and an epithelized tract that communicates between the anorectal lumen and the perineal or buttock skin. [1]. The cumulative incidence of anal fistulas in patients with CD 20 years after diagnosis is 50% with an ~75% of these cases being complex anal fistulas [1]. Complete healing routinely requires several medical and surgical treatments dependent on fistula location, internal and external sphincter involvement and precipitating factors [2]. Morbidity in patients is made worse by scarring, drainage, abscess formation, pain, fecal incontinence and frequent relapses that contribute to stress, depression and a decrease in quality of life [3–5]. We present a patient with complex recalcitrant trans-sphincteric, supra-sphincteric and extra-sphincteric atypical fistulizing perianal CD that was refractory to initial treatment of multiple courses of antibiotics, various biologics and seton placement; he ultimately required fecal diversion prior to achieving partial improvement in symptoms.

## CASE PRESENTATION

**Figure 1:**
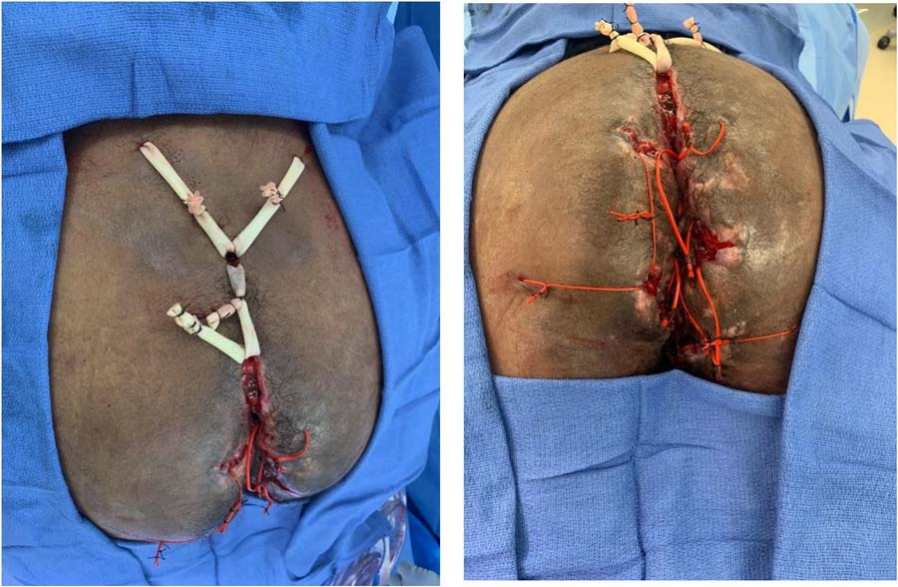
Multiple noncutting setons placed to treat extensive complex and severe trans-sphincteric, supra-sphincteric and extra-sphincteric fistulizing perianal CD featuring multiple external openings, atypical locations and associated perianal abscesses.

A 22-year-old male with a past medical history of hidradenitis suppurativa (HS), malnutrition and recently diagnosed CD presented to our tertiary academic center with extensive trans-sphincteric, supra-sphincteric and extra-sphincteric perianal fistulas. He had fistulizing disease with multiple external openings, atypical locations and perianal abscesses that required several incision and drainage procedures and seton placements prior to presentation at outside institutions. On examination, these openings extended to his mid-back in addition to the perineum. We initially treated him by obtaining source control (drainage of abscess), draining setons and subcutaneous fistulotomies (Fig. 1). After his initial placement of nearly 10 draining setons and three subcutaneous fistulotomies with incision and drainage at our institution, the patient ultimately completed three doses of infliximab but was found to have antibodies afterward, eliminating the possibility of salvage therapy. He was subsequently started on steroid therapy for symptomatic relief. Finally, the gastroenterology and dermatology services agreed that Adalimumab was the most appropriate biological to target both the patient’s CD and HS. Complications with insurance and loss of follow-up both contributed to a delay in initiating further biologic treatment with the patient not starting Adalimumab until several months after his index placement of setons with subcutaneous fistulotomies.

In the 8 months following his index surgery, the patient was readmitted on several occasions due to his extensive fistulizing perianal CD. A reluctance to undergo fecal diversion, issues with treatment compliance, loss of follow up and insurance authorizations contributed to the challenges in managing these complex anal fistulas that would eventually require multiple courses of antibiotics. Multispecialty care was guided by infectious disease consultations, use of biologics (Infliximab, Adalimumab and Ustekinumab), interventional radiology drainage, bowel regimen, pain management, surgical nutrition and physical and occupational therapy. The patient eventually agreed to a laparoscopic loop sigmoid colostomy after further incision and drainage of his perianal abscess, subcutaneous fistulotomy and seton placement.

At the 3-month follow up, the patient was no longer on biologic therapy but endorsed only minor improvement in symptoms. Abdomino-perineal resection had been discussed as a potential future surgical option and the patient remains currently on Ustekinumab therapy.

## DISCUSSION

CD patients frequently require surgical intervention despite recent advancements in biologics [6]. Patients with CD often have aggressive fistulizing perianal phenotypes that result in higher rates of surgery, hospitalizations and corticosteroid dependence as compared with the general population [6]. Unfortunately, there is a lack of convincing data on fistulizing CD; medical and surgical approaches can vary widely in clinical practice [7, 8, 9]. An aggressive approach to complex anal fistulas involving the sphincters may increase the risk of fecal incontinence; these fistulae often persist or recur if primary tracts and secondary extensions are not fully drained [9–11]. Conversely, seton treatment alone is associated with a fistula recurrence rate of up to 47%. In CD patients who have undergone fistulotomies after failure of medical management, fecal incontinence is reported in ~60% of patients [7].

The combination of noncutting setons and medical management has demonstrated complete or partial healing in two-thirds of patients [5]. As our patient did not respond to these measures, he ultimately underwent a loop sigmoid colostomy to effectively divert stool away from his healing tissue. A colostomy can be beneficial for patients with uncontrolled perineal sepsis by diverting stool to minimize soiling and trauma to the operated tissues [6]. In patients who had undergone temporary fecal diversion for refractory anorectal CD, 63.8% of them reported improvement in symptoms within 3–6 months; however, bowel continuity was only attempted in ~35% of patients thereafter [12].

A combination of surgical and medical treatment can yield complete fistula remission in 62% of patients as compared with 43% with monotherapy [7]. Critically, this also involves coordinating multidisciplinary care across specialists in gastroenterology, infectious disease, nutrition and navigating the complex social and psychological barriers that are common in patients with poor access to care. In this case, the dermatology service was intimately involved as his history of HS complicated his fistulizing CD. Despite these efforts, our patient only endorsed minor improvement at his most recent visit and still suffered from significant pain. Abdominal perineal resection may represent one of the last options for treatment in these patients as it is indicated in those with significant perineal disease with associated damage to the sphincter muscles and accompanying fecal incontinence [7]. This case reflects the challenges associated with the care of fistulizing perianal disease for CD patients; management of extensive and complex perianal fistulas in Crohn’s patients requires a staged and multidisciplinary approach with focus on minimizing contamination, treating infection and use of setons, wound care and nutrition.
